# Change in plasma sirtuin 1 level by injection into uterus of resveratrol in Korean cattle

**DOI:** 10.21451/1984-3143-AR2019-0090

**Published:** 2020-03-17

**Authors:** Dae Hyun Kim, Jun Koo Yi, Jae Jung Ha, Dong Yep Oh, Do Yeoung Kim, Yoon seok Lee

**Affiliations:** 1 Livestock Research Institute, Gyeongsangbuk-Do, Yeongju, Gyeongbuk, Republic of Korea; 2 Department of Biotechnology, College of Agriculture & Life Science, Hankyong National University, Gyeonggi, Republic of Korea; 3 Center for Genetic Information, College of Agriculture & Life Science, Hankyong National University, Gyeonggi, Republic of Korea

**Keywords:** mitochondrial biogenesis, embryonic development, resveratrol, plasma SIRT1, Korean cattle

## Abstract

Bovine embryonic development is closely associated with mitochondrial biogenesis, which is regulated by the sirtuin 1 (SIRT1). Resveratrol, which is a type of natural phenol produced by several plants and used as a dietary supplement, is the activator of SIRT1. Although it has been reported that resveratrol increased SIRT1 level in *in vitro* bovine blastocysts, there are no *in vivo* reports on the change in the plasma SIRT1 level in cows. Therefore, we investigated the change in the level of plasma SIRT1 by injecting different concentrations of resveratrol into the uterus of Korean cattle heifer. The level of plasma SIRT1 in the 1.0 μM resveratrol-injected group was the highest among all groups (*P* < 0.05). Although the level of plasma SIRT1 increased on days 7, 9, and 14 in the resveratrol-injected group, the level of plasma SIRT1 in the control group decreased. When 1.0 µM resveratrol was directly injected into the uterus of cows during artificial insemination, a pregnancy rate was 21.0% higher than that in the control group. In conclusion, our results identified that the level of plasma SIRT1 was increased by direct injection of resveratrol and improved conception rate by injection into uterus of cow during artificial insemination.

## Introduction

Recently, several studies have reported that resveratrol, a type of natural phenol used as a dietary supplement, has an effect on oocyte maturation and embryonic development in heifers (Baur and Sinclair, 2006; Gut and Verdin, 2013; Galeati, 2015). Bovine oocyte maturation and embryonic development are closely associated with mitochondrial biogenesis in the oocyte and embryo. The mitochondrial quantity is regulated by the sirtuin 1 (SIRT1) and peroxisome proliferator-activated receptor gamma coactivator 1-alpha (PGC-1α). Resveratrol, which is an activator of SIRT1, binds to the N terminus of the SIRT1 protein, resulting in deacetylation and activation of transcription cofactor PGC-1α. Thus, the mitochondrial number in the oocyte and embryo is increased by PGC-1α (Verdin, 2007; Lavu et al., 2008; Fullerton and Steinberg, 2010; Gut and Verdin, 2013).

Especially, Concentration of 1.0 μM resveratrol enhanced bovine oocyte maturation and post-*in vitro* fertilization embryonic development, probably in a dose dependent manner on SIRT1 (Wang et al., 2014). Furthermore, Pretreatment of bovine embryos with resveratrol improved the quality of embryos through mitochondrial biogenesis (Hayashi et al., 2018). Although it has been reported that resveratrol increased the SIRT1 level in *in vitro* bovine blastocysts, there is no *in vivo* report on the change in the plasma SIRT1 level in heifers. Therefore, the aim of this study was to investigate changes in the level of plasma SIRT1 when injected with different concentrations of resveratrol (0, 0.5, 1.0, and 2.0 μM) into the uterus of Korea cattle heifers.

## Material and methods

### Animals

The animal experiment was approved by the IACUC (Institutional Animal Care and Use Committee) of the Gyeongsangbuk-do Livestock Research Institute and all applicable national laws and policies regarding the care and use of animals were observed during the experiment. Twenty cows (13.8 ± 0.2 years old, 0 parity, 259.4 ± 5.0 kg) were used for analysis of plasma SIRT1 level in this study. Fifty-nine cows (59.2±4.9 years old, 2.9±0.3 parity, 409.4±11.5 kg) were used for pregnancy test in this study. During the experiment, heifers were housed in a stanchion barn with sufficient space and were given feed according to the Korean feeding standards program. Rice straw, mineral blocks, and water were fed *ad libitum*. At the beginning of the experiment, the cows had a mean body condition score of approximately 2.3 ± 0.03 (scale 1 to 5 according to the Korea Animal Improvement Association’s Guide). Abnormalities in the ovaries and uterus detected by transrectal ultrasonography were not selected in this study.

### Analysis of plasma sirtuin 1 level

**Figure 1:**
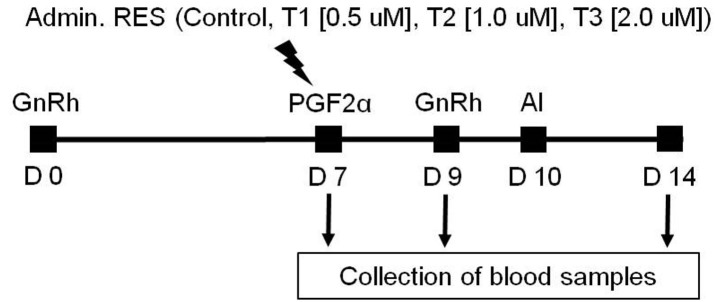
Schematic diagram of ovum synchronization used in this study. The blood sample was collected at 7, 9, and 14 days by a vacuum tube containing EDTA. D: Day, Admin. RES: administration of resveratrol in a dose-dependent manner.

To investigate the level of plasma SIRT1 by resveratrol administration in Korean cattle cows (n = 20), the experimental program was designed based on the ovulation synchronization protocol (Oktay and Haci, 2014). The resveratrol treatment in the program of ovulation synchronization protocol is shown in Figure 1. On day 7, a control group of Korean cattle heifer (n = 5) was injected with 20 ml of PEG400 (diluted 1:1000 with 0.9% physiological saline), whereas three treatment groups (n = 5) were each injected with 20 ml of 0.5, 1.0, or 2.0 μM resveratrol solution (diluted with 0.9% saline) into the uterus. Blood samples were collected on days 7, 9, and 14 from all groups according to the program of ovulation synchronization protocol. The analysis of SIRT1 was performed using Bovine Sirt1 ELISA Kit (MBS103166, MyBioSource INC.).

### Pregnancy

To confirm the conception rate by resveratrol administration in Korean cattle cows (n = 59), the experimental program was the same as analysis of plasma sirtuin 1 level. The resveratrol treatment in the program of ovulation synchronization protocol is shown in Figure 1. On day 7, a control group of cows (n = 28) was injected with 20 ml of PEG400 (diluted 1:1000 with 0.9% physiological saline), whereas treatment groups (n = 31) were each injected with 20 ml of 1.0 μM resveratrol solution (diluted with 0.9% saline) into the uterus. The artificial insemination was performed on Day 10 and blood samples were not collected. Pregnancy test was determined Day 50 using transrectal ultrasonography (HONDA HS-101V, HONDA, Zapan).

### Statistical analysis

The relationship between the level of SIRT1 and resveratrol administration was analyzed using ‘mood’s median test’ in R version 3.5.3. and the total conception rate was analyzed using the Mantele Haenszel procedure. *P* < 0.05 was considered statistically significant.

## Results

**Figure 2:**
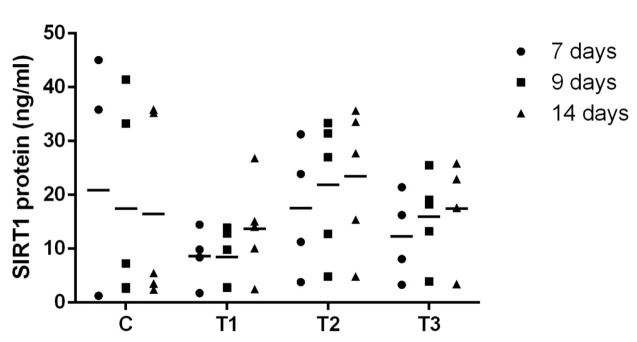
Level of plasma SIRT1 by injection of resveratrol. (C: control, T1: 0.5 µM, T2: 1.0 µM, T3: 2.0 µM resveratrol administration). The bar represents median of plasma SIRT1 concentrations.

We investigated the plasma SIRT1 level with different resveratrol concentrations (0, 0.5, 1.0, and 2.0 μM) injected into the uterus of Korean cattle heifers based on the ovulation synchronization protocol. Change in plasma SIRT1 level in the 0, 0.5, 1.0, and 2.0 μM resveratrol-injected groups on day 7, 9, and 14 based on the ovulation synchronization protocol is shown in Figure 2.

As shown in Figure 2, the level of plasma SIRT1 was increased in the 1.0 μM resveratrol-injected group compared to that in the other groups (*P* < 0.05). Although the level of plasma SIRT1 increased on days 7, 9, and 14 in the resveratrol-injected group, the level of plasma SIRT1 in the control group decreased.

As a result, we investigated the increase in the level of plasma SIRT1 by directly injecting resveratrol into the uterus and it was the highest at 1.0 µM of resveratrol concentration.

When 1.0 µM resveratrol was directly injected into the uterus of cows on day 10 during artificial insemination, we found a higher pregnancy rate (71.0%) than that in the control group (50.0%), (Table 1).

**Table 1:** Pregnancy rates following the injection of resveratrol into the uterus. C: control, T2: 1.0 µM resveratrol administration. a-b, Values with different letters, a and b, are significantly different at P<0.05.

**Group**	**No. of pregnant cows**	**Total**	**Pregnancy rate (%)**
C	14	28	50.0^a^
T2	22	31	71.0^b^
Total	36	59	61.0

C: control, T2: 1.0 µM resveratrol administration. a-b: Values with different letters, a and b, are significantly different at *P*<0.05.

## Discussion

SIRT1 is essential for proper oocyte maturation and embryonic development. Furthermore, resveratrol, which is a protein activator of sirtuin, has an influence on oocyte maturation and embryonic development (Salzano et al., 2014; Takeo et al., 2014). In previous studies, the effect of resveratrol treatment on *in vitro* oocyte maturation and embryonic development has been reported; 0.5 µM resveratrol (Salzano et al., 2014), 1.0 µM resveratrol (Wang et al., 2014; Hayashi et al., 2018), 2.0 µM resveratrol (Takeo et al., 2013). However, there is no report of the *in vivo* effect of resveratrol by injecting resveratrol into the uterus of cattle. It is the best way for measuring the plasma SIRT1 level to identify the effect of resveratrol on pregnancy rates.

As a result of checking the plasma SIRT1 level, the T2 group (1.0 µM resveratrol) increased as shown in Figure 2. Consistent with previous studies, SIRT1 protein level was the highest in the 1.0 µM resveratrol-treated bovine oocyte and resveratrol treatment enhanced SIRT1 expression levels in the blastocysts (Wang et al., 2014; Hayashi et al., 2018). Resveratrol is known to be an SIRT1 activator. The activation of SIRT1 has been postulated to be a key event underlying the biological activities of resveratrol (Singh et al., 2010; Price et al., 2012; Sato et al., 2014). Furthermore, SIRT1 has a role in cumulus expansion, polar body formation and blastocyst hatching rate was improved with pretreatment of resveratrol in bovine embryos (Wang et al., 2014; Hayashi et al., 2018). Especially, pretreatment of blastocysts with 1.0 µM of resveratrol improve the pregnancy rates by the embryo transfer in Japanese black cows (Hayashi et al., 2018).

## Conclusion

In our study, the pregnancy rate was 21.0% higher than in the control group, so upregulation of SIRT1 by directly injecting resveratrol into the uterus could help improve mitochondrial biogenesis. As the result reported by the previous studies, a several research demonstrated that a lower concentration of resveratrol was affected in vitro embryo development and oocyte maturation and improved the pregnancy rates in cow. So, we have performed in vivo experiment and then found that these results are the same results reported by the previous study. Therefore, our results identified that a low resveratrol concentration was increased conception rate by injection into uterus of cow during artificial insemination.
